# Cytocompatibility of Bilayer Scaffolds Electrospun from Chitosan/Alginate-Chitin Nanowhiskers

**DOI:** 10.3390/biomedicines8090305

**Published:** 2020-08-24

**Authors:** Valentina A. Petrova, Alexey S. Golovkin, Alexander I. Mishanin, Dmitry P. Romanov, Daniil D. Chernyakov, Daria N. Poshina, Yury A. Skorik

**Affiliations:** 1 Institute of Macromolecular Compounds of the Russian Academy of Sciences, Bolshoy pr. V.O. 31, 199004 St Petersburg, Russia; valentina_petrova_49@mail.ru (V.A.P.); daniel.chernyakov@gmail.com (D.D.C.); poschin@yandex.ru (D.N.P.); 2 Almazov National Medical Research Centre, Akkuratova st. 2., 197341 St. Petersburg, Russia; golovkin_a@mail.ru (A.S.G.); mishaninssma@yandex.ru (A.I.M.); 3 Institute of Silicate Chemistry of the Russian Academy of Sciences, Adm. Makarova emb. 2, 199034 St. Petersburg, Russia; dprom@mail.ru

**Keywords:** chitosan, alginate, chitin nanowhiskers, electrospinning, tissue engineering, polyelectrolyte complex, biocompatibility, mesenchymal stem cells

## Abstract

In this work, a bilayer chitosan/sodium alginate scaffold was prepared via a needleless electrospinning technique. The layer of sodium alginate was electrospun over the layer of chitosan. The introduction of partially deacetylated chitin nanowhiskers (CNW) stabilized the electrospinning and increased the spinnability of the sodium alginate solution. A CNW concentration of 7.5% provided optimal solution viscosity and structurization due to electrostatic interactions and the formation of a polyelectrolyte complex. This allowed electrospinning of defectless alginate nanofibers with an average diameter of 200–300 nm. The overall porosity of the bilayer scaffold was slightly lower than that of a chitosan monolayer, while the average pore size of up to 2 μm was larger for the bilayer scaffold. This high porosity promoted mesenchymal stem cell proliferation. The cells formed spherical colonies on the chitosan nanofibers, but formed flatter colonies and monolayers on alginate nanofibers. The fabricated chitosan/sodium alginate bilayer material was deemed promising for tissue engineering applications.

## 1. Introduction

The simplicity, cost-effectiveness, and high production rate make electrospinning an advanced and highly relevant technique for the fabrication of nanofibrous scaffolds with unique architecture. The beneficial characteristics of electrospun scaffolds include their high stability and permeability, large surface area, excellent mechanical performance, and ease of functionalization [1,2,3]. These scaffolds therefore have widespread applications in biomedicine as wound dressings, drug carriers, and tissue engineering scaffolds [1,2,3]. When used for cell culture purposes, electrospun scaffolds provide a fibrous and highly porous architecture that mimics the natural extracellular matrix (ECM), thereby stimulating the formation of functional artificial tissues [4]. The 3D features of electrospun scaffolds (fiber diameter, porosity, pore size, and interconnectivity) greatly affect cell behavior, as well as the microclimate and cell viability inside the scaffolds [5]. Electrospun scaffolds can therefore successfully compete with hydrogels, decellularized ECM, and 3D printed scaffolds in the field of tissue regeneration [6].

Electrospinning using biopolymers that decompose naturally produces bioresorbable scaffolds that do not require subsequent surgical elimination. However, pure biopolymers typically have low spinnability with electrospinning techniques developed for synthetic polymers or biopolymer derivatives (e.g., cellulose acetate) [7]. However, many natural polysaccharides, including chitosan (CS), sodium alginate (ALG), and hyaluronic acid [8,9,10], can be electrospun in the presence of a carrier polymer, like polyethylene oxide (PEO). Straightforward electrospinning techniques also permit the use of polymer mixtures or the ready combination of polymers with nanofillers for the development of new functional materials. Nanofillers can also play an important role in the initiation of electrospinning and can lead to advantageous changes in the polymer architecture [11].

Electrospinning proceeds under mild conditions, so drugs (including small molecule drugs, proteins, peptides, and gene vectors [12]) can be easily introduced into the nanofibers while retaining their activity. Electrospinning has also attracted much attention as a method for preparation of bilayer scaffolds that combine the properties of two different polymers. Some polymers undergo natural polyelectrolyte interactions, which means that scaffolds can be obtained with the need for any cross-linking agents [13]. These types of intermolecular interactions can be further promoted by the introduction of nanofillers consisting of mineral (e.g., montmorillonite clay, silica nanoparticles) or organic (e.g., chitin nanowhiskers [CNW]) materials into the polymer matrix. Nanowhiskers derived from chitin or cellulose structures that have amorphous and crystalline regions have unique properties and are promising fillers for the modification of different polymeric materials [14]. CNW have high biocompatibility, are naturally bioresorbable, do not trigger immune responses, and show pronounced antibacterial activity [15,16], making them ideal of many biomedical applications [17,18]. The rod-like morphology of CNW, their intrinsic rigidity, and their strong interfacial interactions give them excellent mechanical performance and thermal and barrier properties [19,20]. CNW have recently been used to fabricate aerogels [21]. 

The high surface area of CNW facilitates their effective interaction with cells, proteins, and other compounds and makes CNW useful in wound dressings [22,23,24]. We have also demonstrated that reinforcement of polymeric composites (films, gels, fibers) with CNW can alter the morphology, structure, and porosity of composites [25] to improve cell adhesion and proliferation [26]. The chemical, morphological, and mechanical properties of CNW make them attractive in tissue engineering as they impart biological and mechanical properties that mimic the native ECM that regulates cell behavior [27,28]. CNW therefore have immense potential in for the development of biocompatible materials. However, the choice of an appropriate model for biocompatibility testing is important. 

Biocompatibility is now considered a property of the whole implant–recipient system [29] and integrates a number of material properties, such as toxicity and immunogenicity. The classical concept of biocompatibility has been largely concerned with implantable medical devices within the human body. However, in vitro cell models are now increasingly recognized as easy screening systems for biocompatibility of new materials and scaffolds [30,31,32]. In tissue engineering, human multipotent mesenchymal stem cells (MMSCs) are particularly promising candidates for use as in vitro biocompatibility models since they are capable of self-renewal and the immunomodulatory activity needed for reconstruction of damaged tissues. MMSCs are multipotent, so they can differentiate into all three cell types: osteoblasts, adipocytes, and chondrocytes [33]. When used in autologous transplantations, they can serve as progenitor cells in the regenerative process and differentiate into specific cell types.

The aims of the present study were to prepare electrospun bilayer scaffolds based on CS and CNW-containing ALG and to study their structure, morphology, and porosity. The adhesion and proliferation of human MMSCs, as a well-established testing model [13,34], were also determined to explore the biocompatibility of the prepared scaffolds.

## 2. Experimental Section

### 2.1. Characterization of Polysaccharides

The chitosan used in this study was from crab shells (Qingdao Honghai Bio-tech Co., Ltd., Qingdao, Shandong, China) and had a viscosity average molecular weight M_η_ of 1.1 × 10^5^ and a deacetylation degree of 0.98 [13]. Sodium alginate with M_η_ of 1.3 × 10^5^ [25] was purchased from Qingdao Bright Moon Seaweed Group Co. Ltd. (Qingdao, Shandong, China). CNW were prepared by partial deacetylation of chitin [35] and had a degree of deacetylation of 0.40 [36]. The CNW size (thickness 6–15 nm, length 100–500 nm) was estimated by scanning electron microscopy [26]. 

### 2.2. Polymer Solutions

The concentrations of the polymer solutions (3% and 4% for CS and ALG, respectively) used in electrospinning were selected to provide uniform spinning. The CS was suspended in water with vigorous stirring for several hours and acetic acid was then added under continuous stirring. Upon complete dissolution of CS, a 3% aqueous solution of PEO was added. The final concentrations of the components were as follows: a 3% solution of CS in 30% acetic acid with the addition of 10 wt% of PEO. ALG was dissolved in water, mixed with a 0.5% aqueous dispersion of CNW (the mass fraction of CNW was 7.5% relative to ALG), followed by addition of PEO under constant stirring to produce a 4% aqueous solution of ALG containing 15 wt% PEO. The calculated amount of 2% acetic acid necessary for protonation of the CNW amino groups was then added to the ALG/PEO solution. All solutions of the starting polymers were purified by filtration under vacuum.

### 2.3. Electrospinning

Electrospinning was performed on a Nanospider NS Lab 500 unit (Elmarco, Liberec, Czech Republic); the distance between electrodes was 22–24 cm; the fibers were collected on a paper substrate on the collector drum. The rotation speed of the spinning electrode was 10–16 min^−1^, the electrospinning voltage was 60–75 kV. The following electrospun materials were obtained: chitosan (CS), ALG with 7.5% CNW (ALG&CNW), and bilayer CS and ALG with 7.5% CNW scaffold (CS-ALG&CNW). 

### 2.4. Film Preparation

The films were prepared using a 3% solution of CS in 2% acetic acid and a 4% aqueous solution of ALG. The films were cast onto a glass substrate using a laboratory spinneret after setting the working air gap (0.6–0.8 mm) with a feeler gauge. The samples were dried in room temperature (RT) air to obtain films with a thickness of 30–40 µm.

### 2.5. General Methods

The rheological properties of the polymer solutions were studied with a Rheotest 2.1 coaxial cylindrical rotational viscometer (Rheotest Medingen GmbH, Ottendorf-Okrilla, Germany) at 20 °C and a shear stress range of 3–600 Pa.

The swelling of the scaffolds in water and in physiological saline (0.9% NaCl) were determined by a gravimetric method. 

Scanning electron microscopy (SEM) studies were performed using a Phenom G Pro scanning electron microscope (Phenom-World BV, Eindhoven, The Netherlands).

Atomic force microscopy (AFM) was performed using a Smena instrument (NT-MDT, Zelenograd, Russia). Samples were scanned in the semi-contact mode; the curvature tip radius was 10 nm, the probe resonant frequency was 173 kHz, and the force constant was 58 N/m. The average fiber diameter was evaluated using ImageJ v. 1.51j8 software (Rasband, W.S., ImageJ, U. S. National Institutes of Health, Bethesda, MD, USA, https://imagej.nih.gov/ij/) by measurement of more than 100 fibers situated mostly perpendicularly to the scan direction. The roughness parameters were calculated using NT-MDT Image Analysis software (NT-MDT, Zelenograd, Russia).

X-ray diffraction was performed with a DRON-3M diffractometer (Burevestnik, St. Petersburg, Russia) using Ni-filtered Cu Kα radiation (λ = 1.5418 Å).

The porosity of the scaffolds was studied using a Porotech 3.1 porosimeter (Porotech Ltd., Vaughan, ON, Canada) and Porovoz software, as described previously [26]. Octane was used as a wetting liquid.

### 2.6. Cell Culture and In Vitro Tests

The cytocompatibility of the scaffolds was evaluated using MMSCs isolated from subcutaneous fat of healthy donors. All in vitro tests were approved by the Almazov National Medical Research Centre Ethics Committee (approval no. 12.26/2014; 1 December 2014) and were performed according to the Declaration of Helsinki. All donors provided written informed consent before subcutaneous fat biopsy. Cells were cultivated in alpha-MEM nutrient medium (PanEco, Moscow, Russia) containing 10% fetal bovine serum (FBS) (HyClone Laboratories, Inc., Logan, UT, USA), 1% L-glutamine, and 1% penicillin/streptomycin (Invitrogen, Waltham, MA, USA) and incubated at 37 °C in a 5% CO_2_ atmosphere. MMSCs from passages 4–5 were used for experiments. The MMSCs were phenotyped using flow cytometry (GuavaEasyCyte8; MerckMillipore, Darmstadt, Germany) and fluorescent anti-CD19, anti-CD34, anti-CD45, anti-CD73, anti-CD90, and anti-CD105 monoclonal antibodies (Becton Dickinson, Franklin Lakes, NJ, USA). CS samples and 12 mm cover glasses were used as the controls for MMSC cell growth. 

Rectangular samples with dimensions of 12 × 8 mm (matching the well size) and cover glasses were treated with 70% ethanol for 10 min, washed three times with phosphate buffered saline (PBS), and placed into a 24-well plate. PBS was added to the samples, and they were left for 24 h. The samples and cover glasses were washed once with PBS, 2 mL of cell medium was then added to each well, and the plate was left for a further 24 h in the CO_2_ incubator to allow uniform distribution of the medium components within the scaffolds. The medium was then removed, 2 mL of MMSC suspension (cell concentration: 5 × 10^4^ cells/mL) was added to each well, and the cells were cultivated in the CO_2_ incubator for 72 h. Cell migration from the plate well surfaces to the material was avoided by transferring the samples and glasses to a new plate on the second day and replacing the cell medium. Each experiment was repeated in triplicate.

After 72 h, the samples and cover glasses were transferred to wells of a new plate, washed with PBS, and fixed with 4% paraformaldehyde for 10 min. After fixation, the samples and glasses were washed with PBS and immunocytochemically stained using antibodies to the cytoskeletal protein vinculin according to standard protocols. Briefly, the samples with adhered cells were treated using Triton X-100 for 10 min, washed three times with PBS, blocked with 1% FBS solution for 30 min at RT, and incubated with a mouse anti-vinculin antibody (Thermo Fisher Scientific, Waltham, MA, USA) at 1:200 dilution for 1 h at RT. The samples with adhered MMSCs were then incubated with Alexa Fluor 568 secondary goat anti-mouse IgG (Invitrogen, Waltham, MA, USA) (1:1000 dilution) for 1 h at RT in the dark. The cells were then stained by incubation with 4,6-diamidino-2-phenylindole (DAPI; dilution 1:40,000) for 40 s. Each incubation procedure was followed by three washes with PBS. Stained samples were stored in PBS in the dark at 4 °C. Cover glasses with cells from control wells were mounted onto microscope slides using a suitable mounting medium and stored in the dark at RT. 

Stained MMSCs on the sample surfaces were visualized with an Axiovert inverted fluorescence microscope (Zeiss, Jena, Germany) equipped with a Canon camera. Images were processed with ZEN software. Pieces of cell-containing material were placed between two microscope slides. DAPI fluorescence was registered using the corresponding filter, while Alexa Fluor 568 fluorescence was registered using the rhodamine channel.

Images of each sample were captured from 10 different fields of view at magnifications of ×10 and ×40. During qualitative analysis, the average size of spheroids was determined from the maximum longitudinal size of 20–30 colonies for every sample. The numbers of cell nuclei stained with DAPI were not calculated, since precise determination of the number of nuclei in the spheroidal colonies on the sample surface was not possible. The obtained data were statistically analyzed using the GraphPad Prism software and the Mann–Whitney non-parameter U-criterion. The results were presented as means and standard deviations (SD).

## 3. Results and Discussion

### 3.1. Preparation and Morphology of CS and CS–ALG&CNW Scaffolds

**Figure 1:**
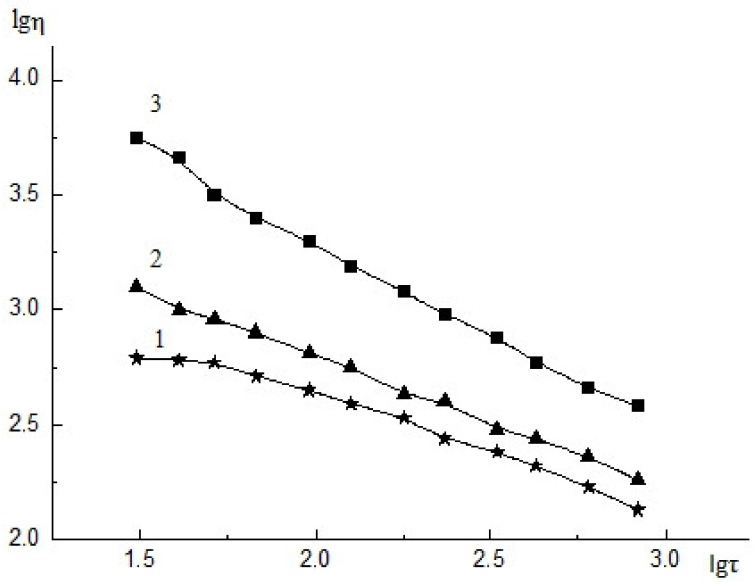
Flow curves of 4% ALG solution (1), 4% ALG solution with 2.5% CNW, 4% ALG solution with 7.5% CNW, 20 °C, η–viscosity (mPa∙s^−1^), τ–shear stress (Pa). The relative standard deviation of the measurements did not exceed 5% (*n* = 3).

The amount of CNW used for modification of the ALG solution was selected experimentally and depended on the viscous properties of the composite solution and its structure. As shown earlier, insoluble CNW particles caused structurization of the CS solution and facilitate electrospinning [37]. The CNW introduced into the ALG solution promoted electrospinning while simultaneously acting as an active filler (due to electrostatic interactions with ALG). Introducing 2.5% CNW into the ALG solution led to an insignificant increase in the viscosity of the composite solution and in the degree of structurization of this solution (Figure 1(2)) compared with that of the initial ALG solution (Figure 1(1)). Increasing the CNW content in the ALG solution up to 7.5% resulted in an increase in the initial viscosity of the solution (Figure 1(3)) and the degree of structurization of the composite solution (due to interactions between the positively charged CNW amino groups and the negatively charged carboxyl groups of ALG). A further rise in the amount of CNW led to a dramatic increase in the solution viscosity and its degree of structurization, and the solutions were no longer suitable for electrospinning.

The electrospinning parameters described in Section 2.3 (voltage of 60–75 kV, distance between electrodes of 22–24 cm, and rotation speed of spinning electrode of 10–16 min^−1^) were experimentally selected for each solution to ensure spinning stability and uniformity of the material. 

**Figure 2:**
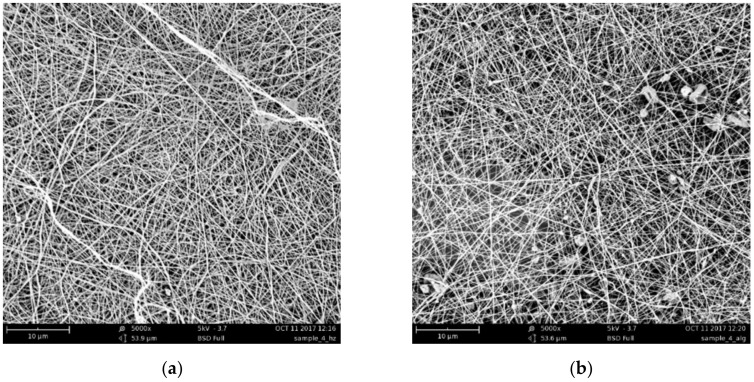
SEM images of the CS surface (**a**) and the ALG&CNW surface (**b**) of the bilayer CS–ALG&CNW scaffold.

The SEM images of the CS–ALG&CNW bilayer scaffold (Figure 2) demonstrated a reasonably uniform fiber formation without any bead formation. The introduction of CNW had a beneficial effect and facilitated the formation of a more uniform material similar to that obtained with CS spinning. The average diameters of the fibers ranged from 360 to 420 nm for the CS side and from 200 to 300 nm for the ALG&CNW side. The distribution of the nanofiber thicknesses was narrower on the ALG&CNW side of the scaffold. Deposition of an ALG&CNW layer onto the CS layer led to the formation of a stable composite at the expense of the interaction between the polyelectrolyte layers.

**Figure 3:**
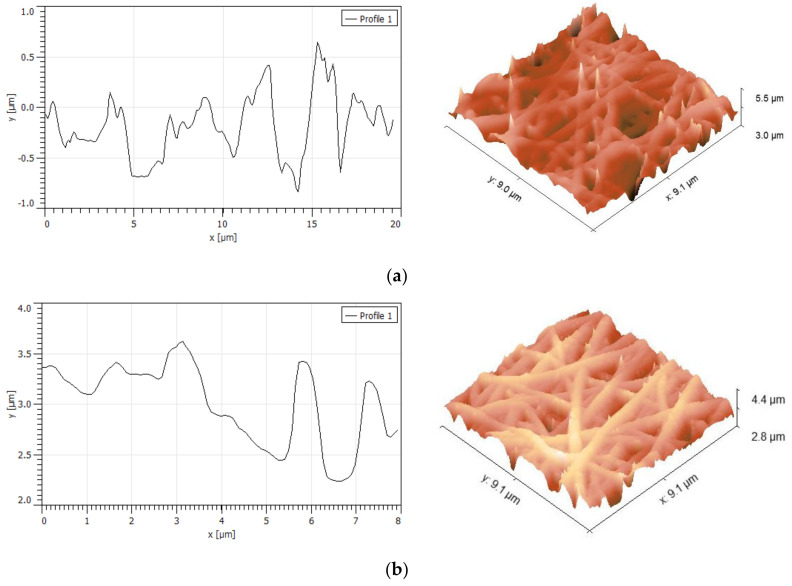
AFM morphology and roughness profiles of the CS surface (**a**) and the ALG&CNW surface (**b**) of the of the bilayer CS–ALG&CNW scaffold.

AFM analysis of the CS–ALG&CNW scaffold also revealed smooth beadless fibers (Figure 3) that had a continuous 3D network structure with interconnected pores similar to those of natural ECM. No significant differences were noted in the surface morphology of the two layers. The average fiber diameter determined from AFM images was 425 ± 58 nm for CS surface and 411 ± 67 nm for ALG&CNW surface. The differences in these results from the SEM measurements for ALG fibers may have been related to an overestimation of the lateral dimensions by the scanning probe, as well as the smaller number of measurements. The fiber network formed surface pores with diameters up to 2 μm and depths up to 1 μm. The rough scaffold surface interacted with the cells to provide numerous contacts.

The roughness parameters are presented in Table 1. The average roughness was evaluated as 0.39 μm for CS and 0.27 μm for ALG&CNW surfaces. These roughness values were higher than those previously estimated for PCL/CS fibers with diameters of 250–400 nm [38]; the average roughness of the nanofibers increased with decreasing fiber diameter and with increases in the dominant alignment direction. However, for electrospun microfibers, the roughness ranged from 1.5–6.3 µm and was dependent on the processing conditions and fiber diameter, but independent of the polymer used [39]. Furthermore, the pore parameters and surface roughness were the main factors that influenced the contact angle and scaffold surface properties [40]. The roughness parameters estimated in this study were typical for electrospun matrices, and the cells responded well to scaffold roughnesses of 0.4–2 μm [41].

### 3.2. X-Ray Diffraction Structure

**Figure 4:**
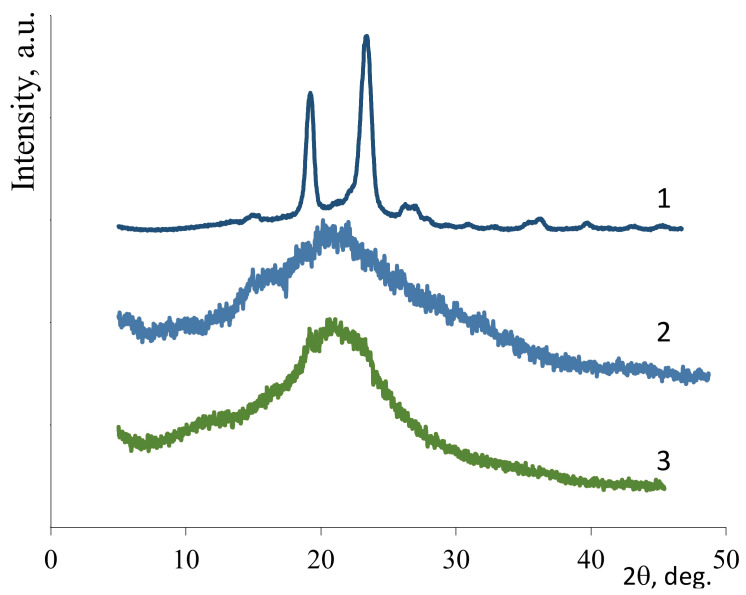
X-ray diffraction patterns: PEO-nonwoven (1), CS-film (2), CS–PEO-nonwoven (3).

**Figure 5:**
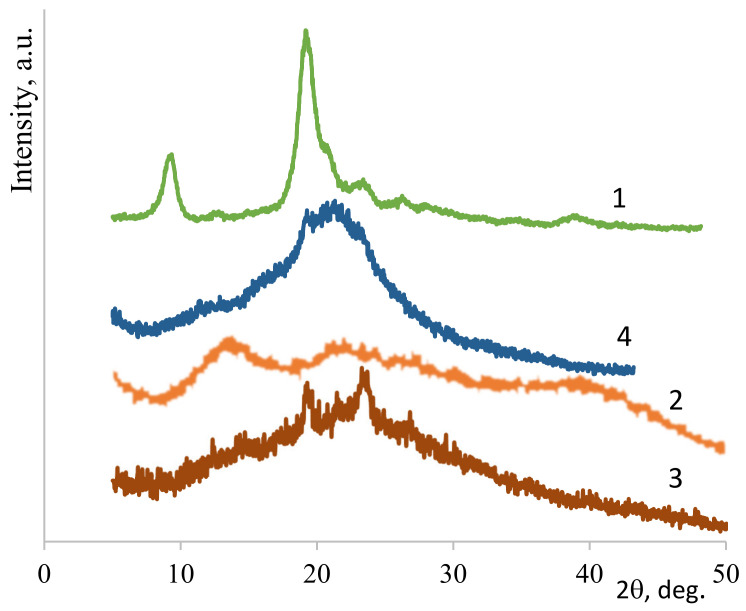
X-ray diffraction patterns: CNW (1), ALG-film (2), ALG&CNW-nonwoven (3), and CS–ALG&CNW-nonwoven (4).

Efficient electrospinning of polysaccharides required the presence of PEO. The nonwoven PEO had a high degree of crystallinity (85%, see Figure 4(1)). As shown previously [13], comparative X-ray diffraction analysis of the polysaccharide films with and without PEO revealed that the interaction between polysaccharides and PEO became stronger during electrospinning. Thus, in the case of nonwoven CS (Figure 4(3)), the signal was more intense in the 2θ range of 19–24° than in the X-ray pattern of the CS film (Figure 4(2)). This finding supported the presence of PEO and an interaction between CS and PEO arising during electrospinning, as confirmed by the literature data [42]. The presence of PEO and CNW in the nonwoven ALG&CNW (Figure 5(3)) led to some changes in its structure when compared with that of the ALG film (Figure 5(2)). The X-ray diffraction pattern of the bilayer CS–ALG&CNW scaffold (Figure 5(4)) included a strong signal in the range 2θ = 19–24° and a shoulder in the range 2θ = 13–15°, which combined the diffraction patterns of CS (Figure 4(2)) and ALG&CNW (Figure 5(3)). This result indicated that strong intermolecular interactions arose during electrospinning and led to changes in the hydrophilicity of the bilayer scaffold, as evidenced by the absence of the reflex 2θ = 9.8° characteristic for *α*-chitin (Figure 5(1)).

### 3.3. Swelling

The bilayer CS–ALG&CNW scaffold consisted of a layer of CS and a layer of ALG&CNW. In addition, a water-insoluble polyelectrolyte complex (PEC) formed during contact of the oppositely charged polymers in the process of multilayer spinning. The swelling of the bilayer scaffold (Table 2) was determined from the properties of the initial polymers and the changes that occurred during electrospinning and subsequent processing. The strong intermolecular interactions between CS and PEO that arose during electrospinning caused a partial loss of solubility in water of nonwoven CS [42,43]. Subsequent heating of the nonwoven CS led to a solubility loss and a reduction in the degree of swelling in water and in physiological saline (Table 2); these changes were associated with the formation of amide crosslinks [44]. The nonwoven ALG&CNW was partially dissolved in water and contained microgels (Table 2) composed of the PEC that formed as a result of the interaction between the CNW amino groups and the ALG carboxyl groups [25]. Heating of the nonwoven CS-ALG&CNW (Table 2) led to a loss of solubility in water and physiological saline due to strengthening of the chemical interactions. 

### 3.4. Porosity

The electrospun monolayer CS [13] and bilayer CS-ALG&CNW scaffolds had large inner surfaces (Table 3) and well-developed pore structures that could facilitate cell adhesion, migration, proliferation, and differentiation. The porosity was somewhat greater for the monolayer CS scaffold than for the bilayer CS-ALG&CNW. The pores were wider in the CS-ALG&CNW scaffold.

### 3.5. Testing of Cell Adhesion Properties

The MMSCs visualized on the sample surfaces formed three groups that differed in their morphological characteristics: adhered cells (isolated, multiple), colonies formed by these cells (monolayer, spheroids), and conditionally non-adhered cells (isolated, multiple) that had presumably undergone apoptosis (Table 4). 

**Figure 6:**
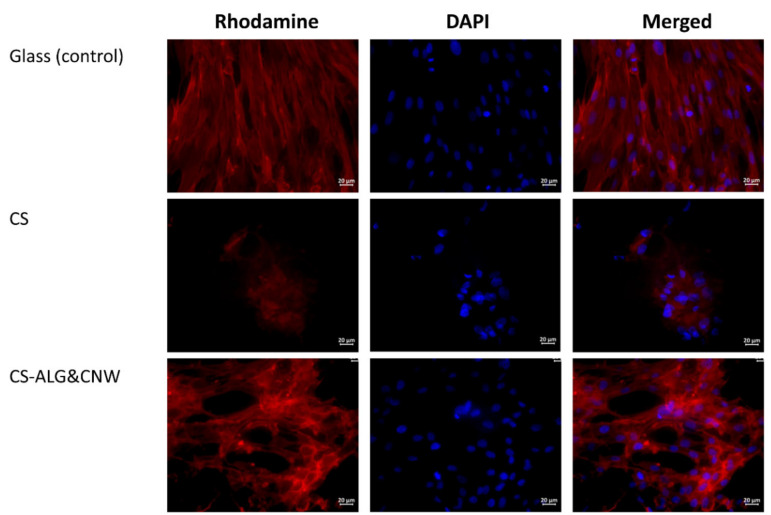
Multipotent mesenchymal stem cells (MMSCs) adhered onto the surfaces of cover glasses and scaffolds. Vinculin in the cell cytoskeletons was stained with a fluorochrome (rhodamine channel); cell nuclei were stained with 4′,6-diamidino-2-phenylindole (DAPI). Combined two-channel image. Magnification ×40.

**Figure 7:**
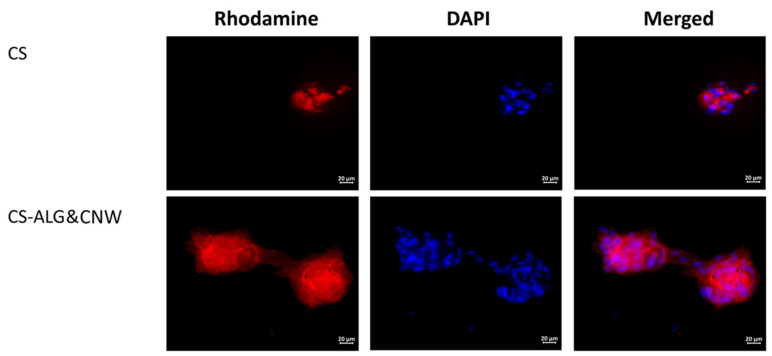
Multipotent mesenchymal stem cell spheroids on sample surfaces. Vinculin in the cell cytoskeletons was stained with a fluorochrome (rhodamine channel); cell nuclei were stained with 4′,6-diamidino-2-phenylindole (DAPI). Combined two-channel image. Magnification ×40.

The cells grown on cover glasses (the control group) were spread on the glass surface and formed a confluent/subconfluent monolayer. They had the typical elongated cell shape with multiple pseudopodia. Many cells were in the process of division (Figure 6). Cells on the CS control sample were located on the surface as individual isolated cells or they formed two-dimensional structures and spheroid colonies of different sizes. The isolated cells had circular shapes. The cells located at the periphery of the large spheroids had a spindle-like shape and gave way to small flat colonies (Table 4, Figure 6 and Figure 7).

The cells on the CS-ALG&CNW scaffold were located on the surface in the form of a monolayer (confluence 70–100%) with predominantly flat colonies and large numbers of spheroids. Cells in the flat colonies had a typical elongated shape with multiple pseudopodia. Isolated cells had circular shapes with small peripheral protrusions. The cells located at the periphery of the spheroids had a spindle-like shape and few pseudopodia. Several spheroidal colonies were connected via “bridges” of cells migrating from the spheroids (Table 4, Figure 6 and Figure 7).

Cultivation of MMSCs on the surfaces of scaffolds based on CS and its composites were characterized by an organization of colonies in the shape of multicellular spheroidal aggregates [13,45]. The formation of spheroids on the surface of CS scaffolds included several stages. The first stage consisted of adhesion and spreading of the cells on the scaffold surface, with formation of multiple pseudopodia. The pseudopodia were then retracted and the cells acquired circular shapes and formed three-dimensional spheroidal colonies [46,47]. Under favorable conditions, the cells located on the periphery acquired spindle-like shapes and migrated onto the adhesive surface of the material via the formation of cellular “bridges” (which interconnected the spheroids with each other). Alternatively, they formed isolated two-dimensional colonies (monolayers with various degrees of confluence). No MMSC migration occurred from the spheroids on non-adhesive polymer surfaces [13,48]. 

The sizes of the spheroids and the degree of confluence of the monolayer consisting of migrating cells were determined by the mobility of the cells in the spheroid aggregates. Cells with high mobility produced larger spheroids more quickly, and they formed two-dimensional colonies with larger areas. An increase in spheroid size was also possible at the expense of merging (for example, on a CS-HA surface). No merging of spheroids was observed on CS scaffolds [46]. Cell mobility depended on the MMSC source and on the adhesive characteristics of the polymer surface, which were determined by the composition of the material. The spheroids formed on CS-HA scaffolds were also larger than those formed on CS [46].

The MMSCs found in spheroids possessed a greater capacity for differentiation. They also showed more pronounced anti-inflammation, regenerative, and reparative properties than those formed in two-dimensional culture [45,48,49]. These features of MMSCs formed in three-dimensional colonies could be explained by the increased expression of genes associated with hypoxia, angiogenesis, inflammation, stress responses, and redox signaling [45].

## 4. Conclusions

The method of scaffold preparation by electrospinning generated fine fiber structures of high porosity and low density that closely mimicked the nature of the ECM and created a good microenvironment for growth of cells and tissues. Polysaccharides (CS, ALG, chitin) were used to develop bilayer electrospun CS–ALG&CNW membranes with average fiber diameters of 200–400 nm and high porosity. ALG electrospinning benefited from the addition of CNW, which provided structuring for the ALG solution. The result was the production of beadless fibers that were able to maintain their structure under high swelling conditions. The pore size of the ALG fibers was larger than that of a CS layer electrospun under the same conditions. Thus, the chemical composition, as well as the layer structure, was favorable for cell growth. 

In vitro testing of the monolayer CS scaffold and the bilayer CS–ALG&CNW scaffold revealed spheroidal MMSC colonies on the surfaces of both types of samples. Indirect signs of cell migration from the spheroids (spindle-like cells with pseudopodia on the periphery of the spheroids) were observed on the surfaces of both samples. Some of the spheroids on the CS–ALG&CNW scaffold were connected to each other via “bridges” of migrating cells. These cell “bridges” that formed between the spheroids on the CS–ALG&CNW scaffold were flat monolayer cultures with a confluence reaching 100% in some areas. The wide surface area of these flat colonies was possibly a result of their primary formation from a portion of adhered cells, rather than from migration and proliferation of cells from the spheroids. The obtained data indicate that the deposition of an outer ALG layer modified with 7.5% CNW onto the surface of a CS-based composite material improved the adhesive characteristics of the scaffold. This could indicate an increased biocompatibility of the CNW-modified scaffolds.

## Figures and Tables

**Table 1:** Roughness parameters of CS and ALG&CNW surfaces of the bilayer CS–ALG&CNW scaffold.

Roughness Parameter	CS Surface	ALG&CNW Surface
Peak-to-peak roughness, µm	6.13	4.76
Average roughness, µm	0.39	0.27
Root mean square roughness, µm	0.50	0.25

**Table 2:** Swelling of nonwoven materials (mean ± SD, *n* = 3).

Sample	Treatment	Swelling in Water, g/g	Swelling in 0.9% NaCl, g/g
CS	80 °C, 4 h	5.3 ± 0.3	2.2 ± 0.2
ALG&CNW	-	partially dissolved	partially dissolved
CS-ALG&CNW	80 °C, 4 h	2.5 ± 0.2	2.0 ± 0.2

**Table 3:** Porosity of the scaffolds.

Parameter	CS [13]	CS-ALG&CNW
Average logarithmic pore radius (nm)	1.62	2.26
Average pore radius, nm	489	2193
Porosity over weight, cm^3^/g	9.36	8.48
Porosity over volume, cm^3^/cm^3^	0.976	0.861
Meso- and macro-pore surface over weight, m^2^/g	878	878
Meso- and macro-pore surface over volume, m^2^/cm^3^	89.1	89.1
Total pore surface over weight, m^2^/g	2464	1463
Total pore surface over volume, m^2^/cm^3^	257	149

**Table 4:** Characteristics of multipotent mesenchymal stem cells (MMSCs) and cell colonies formed on different scaffolds at 3 days after seeding.

Sample	Non-Adhered Cells	Adhered Cells	Prevailing Type of Colony	The Average Size of MMSC Spheroids, µm (mean ± SD)
Glass (control)	-	multiple	monolayer	
CS	isolated	multiple	spheroids	70±37
CS-ALG&CNW	isolated	multiple	monolayer + spheroids	109±43*

* Reliability of changes in comparison with the control group (Mann−Whitney): * *p* < 0.05.
